# Mothers

**Published:** 1884-02

**Authors:** 


### MOTHERS.

This book reaches mothers to whom and in behalf of whose children we take pleasure in recommending an excellent and very exceptional medicine. No medicine should be given to a child without the mother's full knowledge of its character. Castoria is a standard prescription of a distinguished physician—old Dr. Pitcher—still living at Hyannis, Mass. A list of its ingredients accompanies each bottle. It is purely vegetable and is not narcotic. It may be given without change of diet or fear of harm, and in its effects is superior to Paregoric, Morphine Syrups or Castor Oil, without the dangerous properties of the former or the nauseating effects of the latter. Of this preparation physicians speak in high terms.

